# Genome-wide analyses using multi-locus models revealed marker-trait associations for major agronomic traits in *Sorghum bicolor*

**DOI:** 10.3389/fpls.2022.999692

**Published:** 2022-10-07

**Authors:** Muluken Enyew, Tileye Feyissa, Anders S. Carlsson, Kassahun Tesfaye, Cecilia Hammenhag, Amare Seyoum, Mulatu Geleta

**Affiliations:** ^1^ Institute of Biotechnology, Addis Ababa University, Addis Ababa, Ethiopia; ^2^ Department of Plant Breeding, Swedish University of Agricultural Sciences, Alnarp, Sweden; ^3^ Ethiopian Biotechnology Institute, Addis Ababa, Ethiopia; ^4^ National Sorghum Research Program, Crop Research Department, Melkassa Agricultural Research Center, Ethiopian Institute of Agricultural Research, Adama, Ethiopia

**Keywords:** candidate gene, genome wide association study, linkage disequilibrium, population structure, quantitative trait locus, sorghum

## Abstract

Globally, sorghum is the fifth most important cereal crop, and it is a major crop in Ethiopia, where it has a high genetic diversity. The country’s sorghum gene pool contributes significantly to sorghum improvement worldwide. This study aimed to identify genomic regions and candidate genes associated with major agronomic traits in sorghum by using its genetic resources in Ethiopia for a genome-wide association study (GWAS). Phenotypic data of days to flowering (DTF), plant height (PH), panicle length (PALH), panicle width (PAWD), panicle weight (PAWT), and grain yield (GY) were collected from a GWAS panel comprising 324 sorghum accessions grown in three environments. SeqSNP, a targeted genotyping method, was used to genotype the panel using 5,000 gene-based single nucleotide polymorphism (SNP) markers. For marker-trait association (MTA) analyses, fixed and random model circulating probability unification (FarmCPU), and Bayesian-information and linkage-disequilibrium iteratively nested keyway (BLINK) models were used. In all traits, high phenotypic variation was observed, with broad-sense heritability ranging from 0.32 (for GY) to 0.90 (for PALH). A population structure, principal component analysis, and kinship analysis revealed that the accessions could be divided into two groups. In total, 54 MTAs were identified, 11 of which were detected by both BLINK and farmCPU. MTAs identified for each trait ranged from five (PAWT and GY) to fourteen (PH) representing both novel and previously identified quantitative trait loci (QTLs). Three SNPs were associated with more than one trait, including a SNP within the Sobic.004G189200 gene that was associated with PH and PAWT. Major effect SNP loci, Sbi2393610 (PVE = 23.3%), Sbi10438246 (PVE = 35.2%), Sbi17789352 (PVE = 11.9%) and Sbi30169733 (PVE = 18.9%) on chromosomes 1, 3, 5 and 9 that showed strong association signals for PAWD, DTF, GY and PALH, respectively, were major findings of this study. The SNP markers and candidate genes identified in this study provide insights into the genetic control of grain yield and related agronomic traits, and once validated, the markers could be used in genomics-led breeding.

## 1 Introduction

Globally, sorghum [*Sorghum bicolor* (L.) Moench] is the fifth most important cereal crop in terms of both production and acreage (FAOSTAT, 2020). Due to its high adaptability to diverse environments, drought tolerance, low input requirements, and high nutritional value, sorghum is considered a vital food security crop (Awika and Rooney, 2004; Dykes, 2019; Jankowski et al., 2020; Abreha et al., 2022). It has various interesting characteristics, including its C4 photosynthesis pathway, drought tolerance, and a relatively small genome that makes it a model crop for cereal genomics (Mullet et al., 2014). The first whole-genome sequence of sorghum was first released in 2009 (Paterson et al., 2009) and the latest version (version 3.1.1) has a genome size of 732.2 Mega base pairs (Mb) (McCormick et al., 2018). Publicly available sorghum genomic sequences provide opportunities for the development of informative molecular markers for different applications, including genome-wide association analyses for the identification of genomic regions associated with complex traits (Girma et al., 2019).

Ethiopia is a center of origin and diversity of sorghum (De Wet and Harlan, 1971), and the Ethiopian sorghum gene pool has been widely used for enhancing desirable traits, such as drought tolerance, resistance to ergot disease and green bugs, and high lysine content (Singh and Axtell, 1973; Borrell et al., 2000; Wu et al., 2006). The gene pool comprises a genetically diverse germplasm that exhibits wide variation in grain yield and other major phenotypic traits such as days to flowering, plant height, biomass, and inflorescence architecture (Amare et al., 2015; Girma et al., 2019; Enyew et al., 2021). The use of such diverse germplasm widely adapted to both biotic and abiotic stresses is essential to understanding the genetics of the target trait variations.

Genome-wide association study (GWAS) is an efficient method to discover genomic regions associated with traits of interest and has been successfully implemented in various crops, including maize (Cui et al., 2016), rice (Zhong et al., 2021), wheat (Alemu et al., 2021) and sorghum (Girma et al., 2019). In GWAS, differentiating true associations from false-positive marker-trait association (MTA) caused by population structure and kinship has been a major challenge (Kaler et al., 2020). Consequently, a number of statistical models have been developed in order to control these spurious marker-trait associations, including a single-locus mixed linear model (MLM) that incorporates these two confounding factors into the analysis as covariates (Price et al., 2006). Nonetheless, this model may lead to false-negative MTAs as result of overfitting, possibly resulting in missed opportunities to uncover loci associated with desirable traits (Liu et al., 2016). To overcome such a false-negative MTA, multi-locus models such as fixed and random model circulating probability unification (FarmCPU) (Liu et al., 2016) and Bayesian-information and linkage-disequilibrium iteratively nested keyway (BLINK) (Huang et al., 2019) have been developed.

The FarmCPU model is a multi-locus linear mixed model (MLMM) designed to eliminate false-positive MTAs without compromising true associations (Liu et al., 2016), by including multiple markers at the same time as covariates to partially eliminate the confounding effect of markers and kinship. It employs both the fixed-effect model (FEM) and random effect model (REM) iteratively in order to completely remove cofounding. Compared to other GWAS models, it provides higher statistical power and is more computationally efficient (Liu et al., 2016). However, FarmCPU is time inefficient when large numbers of markers and individuals are involved. Consequently, BLINK, which has a higher statistical power and is more time-efficient, was recently developed. BLINK reduces computing time by replacing random effect with fixed effect model through approximating maximum likelihood using Bayesian information criterion (BIC) (Huang et al., 2019). Unlike FarmCPU, BLINK uses linkage disequilibrium (LD), thereby removing the assumption that causal genes are evenly distributed across the genome.

Through GWAS, multiple genomic regions associated with different agronomic traits, including grain yield, have previously been identified in sorghum (Morris et al., 2013; Boyles et al., 2016; Girma et al., 2019). However, most of these studies were based on germplasm that have gone through sorghum improvement programs (Morris et al., 2013; Boyles et al., 2016), which reduces the genetic diversity of the crop, as only genotypes bearing desirable traits are selected. For example, breeding to develop early maturing and photoperiod insensitive genotypes excludes late maturing and photoperiod sensitive genotypes (Klein et al., 2008). This limits the ability to detect genomic regions associated with traits of interest through GWAS.

Properly planned and executed GWAS studies often reveal novel genomic regions associated with target traits, thereby facilitating the identification of genes that control those traits. However, because genetic variation exists both within and among different accessions, and genotype-by-environment interactions (G×E) have significant effects on complex traits, multi-environment field trials are crucial to detect stable QTLs through GWAS and validate their robustness in diverse germplasm (Russell et al., 2020). Therefore, this study aimed to conduct GWAS for grain yield and other agronomic traits of 309 diverse Ethiopian sorghum landrace accessions grown across three environments in Ethiopia. Thus, this study aimed at conducting genome-wide association studies (GWAS) for grain yield and other agronomic traits in sorghum, using 309 diverse Ethiopian sorghum landrace accessions, in order to identify novel genomic regions (QTLs) associated with these traits, in addition to confirming those already detected. Furthermore, the study aimed to determine their genetic diversity and population structure, as well as to locate candidate genes within the identified genomic regions.

## 2 Materials and methods

### 2.1 Plant materials

A total of 320 landrace accessions and four accessions of improved varieties were used in this study (**Supplementary Table 1**). Among the 320 landrace accessions, 261 were obtained from Melkassa Agricultural Research Center (MARC) but were originally collected by Ethiopian Biodiversity Institute (EBI) from different geographic regions across different agro-ecological zones. The 59 remaining landrace accessions were collected specifically for this study from farmers’ fields in areas prone to drought in the country. The four accessions of improved varieties (Argiti, Melkam, B35, and ESH4) are drought tolerant and high-yielding and were provided by MARC (**Supplementary Table 1**).

### 2.2 Field trials and phenotyping

Field trials were conducted using the 324 accessions during the main crop-growing season in 2019 at three locations in Ethiopia, Melkassa (MK; 8°24’N, 39°21’E), Mieso (MS; 12°9’N, 7°31’E), and Mehoni (MH; 8°41’N, 39°37’E), which represent different agro-ecological zones (**Supplementary Table 2**). The field experimental design was alpha lattice, comprising 12 blocks with 27 plots in each block. The experiments were conducted in two replications at each of the three sites. The plot size was 2.25 m^2^ (3 m by 0.75 m), and the seeds were planted in a single row of 3 m along the center of each plot. The plants were then thinned out, at a seedling stage, to a spacing of 0.2 m between plants in each plot. The recommended amount of Di-Ammonium Phosphate (DAP) fertilizer (100 kgha^-1^) was applied during planting and Urea (50 kgha^-1^) was applied as a side dressing 40 days after planting. Phenotypic data was collected from five randomly selected and tagged plants in each plot for days to flowering (DTF), plant height (PH), panicle length (PALH), panicle width (PAWD), panicle weight (PAWT), and grain yield (GY) (**Supplementary Table 3** **)**.

### 2.3 Genomic DNA extraction

For DNA extraction and subsequent genotyping of the 324 accessions, seeds harvested from the five phenotyped plants in the first replicate at the Melkasa site were used. The seeds were planted in a greenhouse at the Department of Plant Breeding, Swedish University of Agricultural Sciences (SLU), Sweden. Soon after germination, extra seedlings were removed and only one seedling per mother plant was maintained. The leaf tissue of the five seedlings of each genotype was collected together two weeks after planting, using the BioArk leaf collection kit (LGC Biosearch Technologies). Using a punch with a diameter of 6 mm that was provided with the kit, ten leaf discs (2 leaf discs per plant) were sampled from each genotype and placed in a single well of 96-well plates. Hence, a pool of leaf tissue from five plants were used to represent each of the 324 accessions. The samples were then sent to LGC Genomics (Berlin, Germany) where genomic DNA extraction was conducted. The Sbeadex plant kits were used to extract high-quality genomic DNA (https://biosearch-cdn.azureedge.net/assetsv6/sbeadex-plant-data-sheet.pdf).

### 2.4 SNP selection, seqSNP assay design, sequencing and genotype calling

The 324 sorghum accessions used in the present study were genotyped using SeqSNP, which is an advanced targeted genotyping by sequencing method. Genotyping was conducted using 5,000 SNP markers used in a recently published study on genetic diversity analysis of sorghum accessions (Enyew et al., 2022). The source of the vast majority of the SNPs (93.7%) used was the sorghum SNP database SorGSD (http://sorgsd.big.ac.cn), a web portal that provides genome-wide SNP markers for diverse sorghum genetic resources (Luo et al., 2016). The remaining 6.3% of the SNP markers were identified by aligning functionally annotated sorghum genes with the latest sorghum reference genome using BLAST (Basic Local Alignment Search Tool). The markers are somewhat evenly distributed across the ten sorghum chromosomes and their descriptions can be found in the supplementary material (**Supplementary Tables 2** and **3**) of Enyew et al. (2022); https://www.frontiersin.org/articles/10.3389/fpls.2021.799482/full#supplementary-material).

All the 5,000 markers were designed in a high-specificity assay that does not allow off-target hits against the sorghum reference genome, and are fully covered (two oligo probes were used per target), as described in Enyew et al. (2022). Following the production of the SeqSNP kit, containing the 10K high-specificity oligo probes for the 5,000 SNPs, and the preparation of the sequencing library, the target SNPs were sequenced on an Illumina Nextseq 500/550 v2 system in 75 bp single read sequencing mode. On average, the effective target SNP coverage per sample was 365x. Following sequencing, the quality trimmed reads were aligned to the reference genome using Bowtie2 v2.2.3 (Langmead and Salzberg, 2012), and the SNP genotyping pipeline was set to diploid. The variant identification and genotype calling were done using Freebayes v1.0.2-16 (Garrison and Marth, 2012). In accordance with the standard genotype calling pipeline, allele counts below eight were set to zero before genotype calling to remove SNPs resulting from sequencing errors.

### 2.5 SNP data filtering and format for data analyses

The genotype calling for the 324 sorghum accessions across the 5,000 SNP loci resulted in 84.3% homozygous calls, 14.2% heterozygous calls, and 1.6% missing data. Among the 5,000 SNP loci, 4,695 (86%) were polymorphic whereas 305 loci (14%) were monomorphic across the 324 accessions. Among the 4,695 polymorphic SNP loci, 4,639 were bi-allelic whereas 56 were multi-allelic. Further filtering of the bi-allelic loci was carried out by eliminating 16 accessions that showed heterozygosity in more than 50% of the loci, leaving 309 accessions for further analysis. Among the 4,639 bi-allelic loci, those with missing data above 2%, or with minimum allele frequency (MAF) below 5%, or heterozygosity above 17% were excluded. This resulted in 3,143 high-quality SNP loci, which were used for the analyses of the 309 sorghum accessions.

Data analyses were conducted using diploid genotypes (instead of allele frequencies) since the software packages used were developed for genotypic data analyses, regardless of the fact that each accession was represented by a pool of five genotypes. This approach was considered appropriate because the vast majority of the loci (at least 83%) were homozygous among the 309 accessions, indicating that the five individuals in each accession had the same genotype at those loci. Nevertheless, since both alleles were found at up to 17% of the loci, a genotype in each pool could be homozygous or heterozygous at these loci. The recent individual genotype-based study using these SNP markers revealed only 6.7% (on average) heterozygotes in each accession (Enyew et al., 2022). Thus, the individuals in each pool are mainly homozygotes at these loci. When there is a marker-trait association (MTA), a heterozygote may represent the average phenotypic value of the five plants better than a homozygote. Therefore, all loci containing both alleles were treated as “heterozygous” for the purpose of the data analyses, including the GWAS.

### 2.6 Phenotypic data analysis

Phenotypic data analysis was carried out using the Multi-Environment Trial Analysis with R (META-R) software package (Alvarado et al., 2020). The combined analysis of variance (ANOVA) was conducted by incorporating genotypes, environments, genotype-by-environment interactions (G×E), replications, and blocks as variance components. The META-R was used to calculate the best linear unbiased prediction (BLUP) and to estimate the variance components using the restricted maximum likelihood (REML) method by implementing linear models in lmer function of lme4 package for R. The broad-sense heritability (H^2^) of all traits was also calculated using META-R.

### 2.7 Population structure and linkage disequilibrium analysis

Principal coordinate analysis (PCoA) was used to determine the population structure of the sorghum accessions using GenAlEx 6.5 software (Peakall and Smouse, 2012). A Bayesian clustering algorithm implemented in the STRUCTURE software v.2.3.4 (Pritchard et al., 2000) was used to determine the degree and pattern of population admixture. The STRUCTURE program was run using the admixture model with burn-in periods of 10,000 and a Markov chain Monte Carlo (MCMC) replications of 200,000. The analysis was performed for K ranging from two to ten, with 10 iterations at each K, to determine the optimum number of genetic populations. The optimum K value was predicted following the simulation method of Evanno et al. (2005) using STRUCTURE HARVESTER version 0.6.92 (Earl, 2012).

Genome-wide LD analysis was carried out using Trait Analysis by Association, Evolution, and Linkage (TASSEL) through determining the pairwise squared allele-frequency correlations (r^2^) between SNP markers with sliding window of 50 SNPs. The r^2^ values were then plotted against physical distance to estimate the extent of LD between pairs of loci. The genome-wide LD decay curve line was fitted into the scatterplot using the smoothing spline regression line to estimate the LD decay rate as described by Hill and Weir (1988) in R environment.

### 2.8 Genome-wide association study

GWAS was performed using the statistical genetics package Genome Association and Prediction Integrated Tool (GAPIT) (Tang et al., 2016) within the R environment (Team R.C, 2020). The GWAS was based on genotypic data for 3143 SNP markers alongside phenotypic data comprising six phenological and agro-morphological traits (DTF, PH, PALH, PAWD, PAWT and GY) for 309 sorghum accessions. Principal component analysis (PCA) and pairwise genetic relationship (kinship matrix) according to VanRaden (2008) were calculated through the pipeline implemented in GAPIT. The principal components and the kinship matrix were used to control the population and family structure for GWAS. Two multi-locus GWAS models, BLINK (Bayesian-information and Linkage-disequilibrium Iteratively Nested Keyway) (Huang et al., 2019) and FarmCPU (Fixed and random model Circulating Probability Unification) (Liu et al., 2016) were used to identify significant SNPs for the six traits. The P-value threshold of 0.05 with Bonferroni correction (0.05/number of markers) was used to determine the significant associations for each trait as implemented in GAPIT. Manhattan and QQ plots were generated using the R package qqman (Turner, 2014). The physical map positions of all significant SNPs were determined by aligning their reference sequences to a sorghum reference genome v3.1.1 (McCormick et al., 2018) using JBrowse (Skinner et al., 2009) in Phytozome v12.1 (Goodstein et al., 2012) in order to explore annotated genes within each QTL region.

## 3 Results

### 3.1 Phenotypic variation and heritability of traits

**Figure 1:**
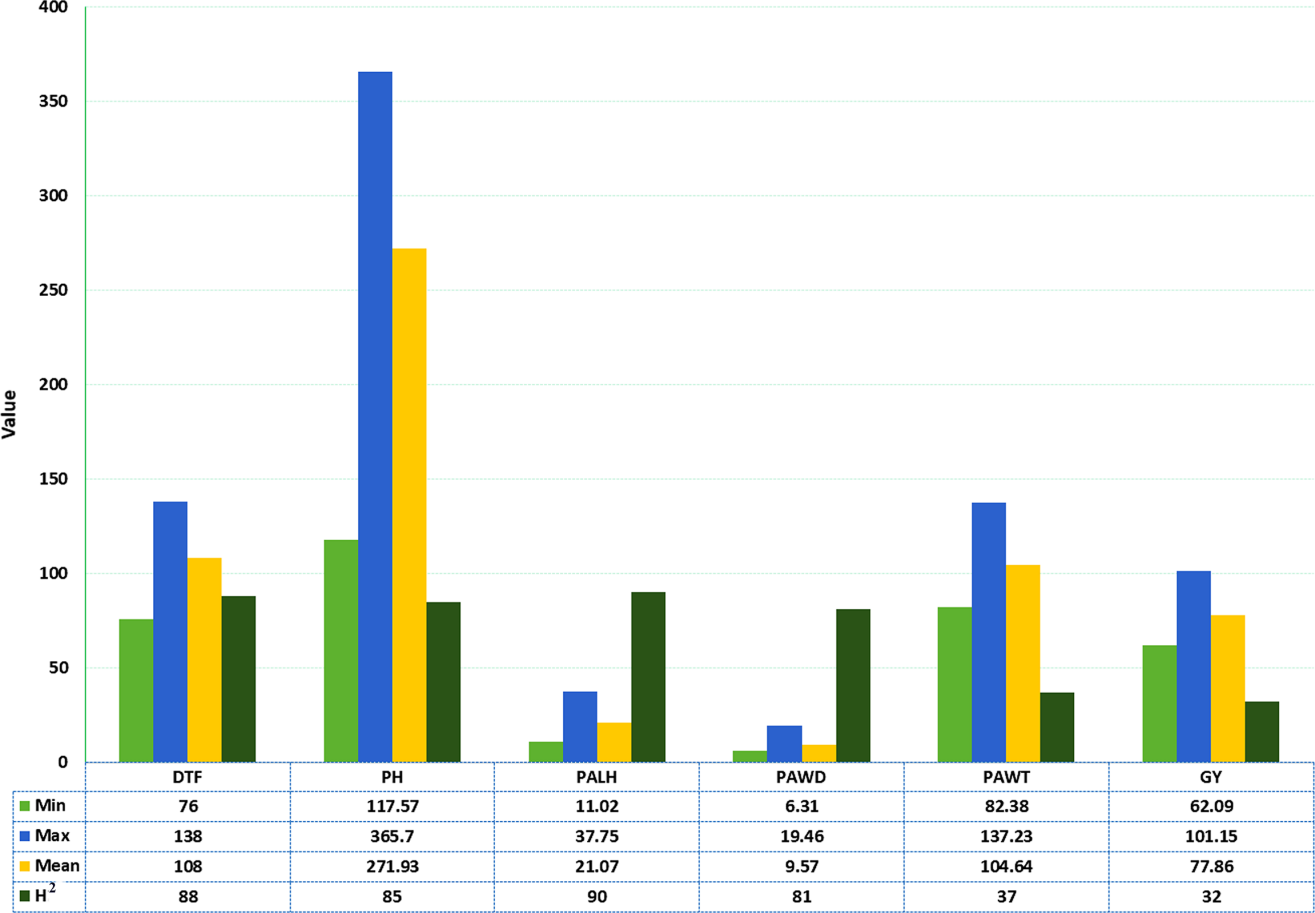
Descriptive statistics and broad-sense heritability of the six phenotypic traits for the sorghum accessions grown at three locations. MIN, Minimum; MAX, Maximum; H^2^, Broad-sense heritability in percent; DTF, Days to flowering; PH, Plant height in cm; PALH, Panicle length in cm; PAWD, Panicle width in cm; PAWT, Panicle weight in g; GY, Grain yield in g.

Large phenotypic variations were observed among the sorghum accessions used in the field trials across three locations for the six phenotypic traits (**Figure 1** and **Table 1**). DTF varied from 76 to 138 days with a mean of 108 days whereas PH varied from 118 to 366 cm with a mean of 272 cm. PALH varied from 11 to 38 cm with a mean of 21 cm, PAWD varied from 6 to 19 cm with an average value of 10 cm. The PAWT for the accessions ranged from 82 to 137 g with a mean of 105 g. The average GY was 78 g, while individual accessions produced 62 to 101 g grain (**Figure 1**). The broad-sense heritability (H^2^) was high for DTF (0.88), PH (0.85), PALH (0.90), and PAWD (0.81), whereas it was moderate for PAWT (0.37) and GY (0.32) (**Figure 1**).

**Table 1:** Combined analysis of variance for grain yield and other target traits for sorghum accessions grown at three locations.

Source	DTF	PH	PALH	PAWD	PAWT	GY
**σ^2^GEN**	151.01***	1817.60***	44.21***	8.16***	253.61***	135.05***
**σ^2^ G×E**	45.61***	389.83***	6.06***	1.49***	805.56***	531.78***
**σ2ENV**	126.86***	27.18^ns^	9.18**	0.36^ns^	1560.13***	753.07**

*** Significant at 0.001 significance level; ** Significant at 0.01 significance level; ns, not significant; GEN, Genotype; ENV, Environment; DTF, Days to flowering; PH, Plant height; PALH, Panicle length; PAWD, Panicle width; PAWT, Panicle weight; GY, Grain yield. σ^2^GEN, genotypic variance; σ^2^G**×**E, genotype-by-environment interaction variance; σ^2^E, environmental variance.

The analysis of variance (ANOVA) revealed that genotypes, environments, and G×E had significant effects on phenotypic variation, except that environments had no effect on PH and PWAD (**Table 1**). Phenotypic variation due to genotypes was higher for DTF, PH, PALH and PAWD compared to that of G×E and environments. In the case of PAWT and GY, environments had higher effects than genotypes and G×E on the phenotypic variation (**Table 1**). For combined environments, a normal frequency distribution was observed for the traits, including grain yield (**Supplementary Figure 1** **).**

### 3.2 Population structure and linkage disequilibrium

**Figure 2:**
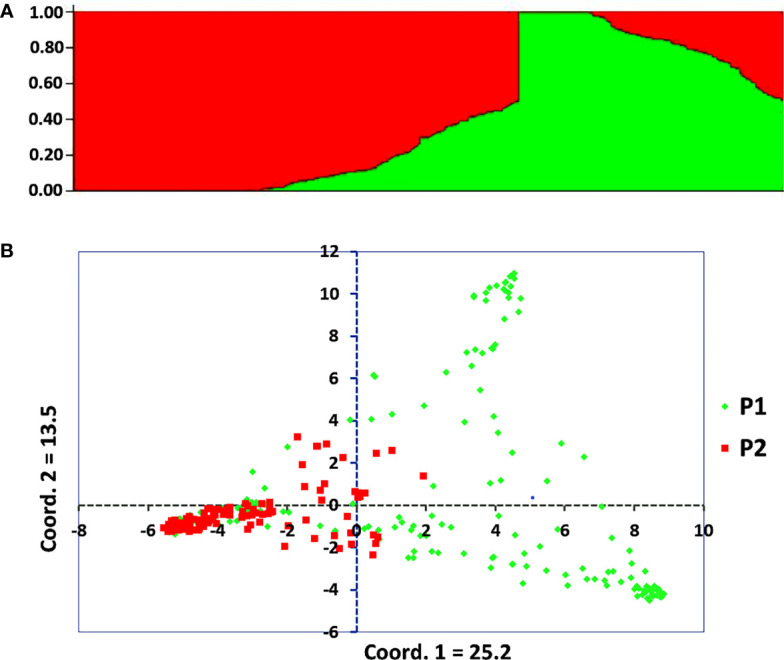
Graphical display of the population structure for 309 sorghum accessions analyzed using genotypic data of 3,143 SNP markers with MAF > 5%. **(A)** Population genetic structure plot of the sorghum accessions for K = 2, and **(B)** PCoA scatter plot depicting the clustering pattern of the 309 individual accessions, with population one (P1) and population two (P2) comprising 121 and 188 accessions, respectively.

**Figure 3:**
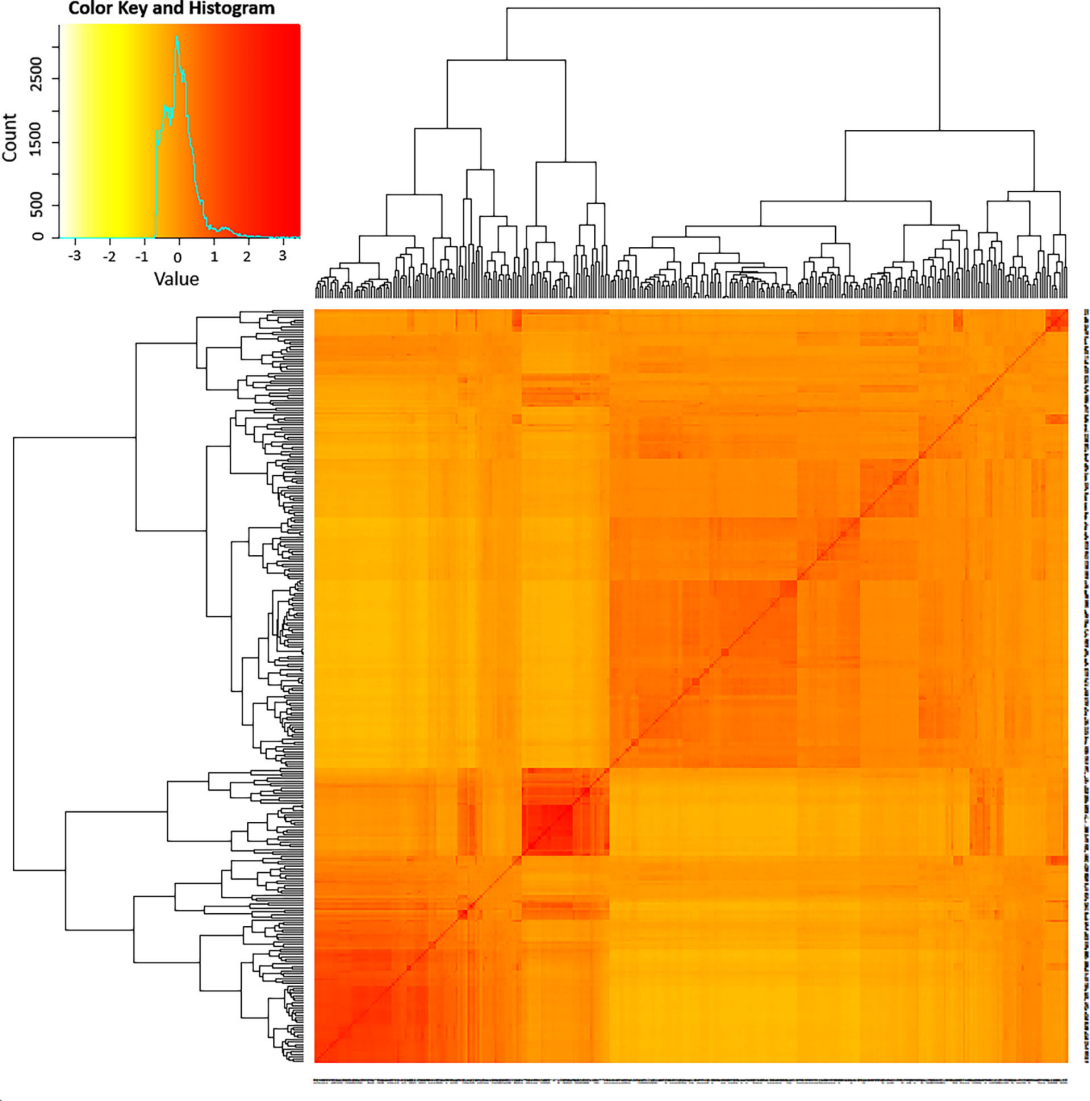
A heat map of kinship matrix illustrating the genetic relationship between 309 sorghum accessions as revealed based on 3143 high quality SNPs with MAF > 5%. The accessions were grouped into two major clusters. The histogram in the color key depicts the number of coefficient values within the corresponding color bar.

The admixture model-based population genetic structure of the 309 sorghum accessions was inferred using STRUCTURE software. The analysis of the STRUCTURE output using STRUCTURE HARVESTER program (Earl, 2012), which implements ΔK method of Evanno et al. (2005) showed that the highest ΔK value was attained at K = 2, indicating that two genetic populations represent the sorghum accessions used in this study (**Supplementary Figure 2**). In this analysis, 121 accessions (39%) were assigned to cluster-I (population one) while 188 accessions (61%) were assigned to cluster-II (population two). As shown in the STRUCTURE plot, some individual accessions possessed alleles inherited from both genetic populations (**Figure 2A**). Furthermore, the principal coordinate analysis (PCoA) revealed a population stratification among the accessions used in agreement with the results obtained with STRUCTURE analyses (**Figure 2B**). The kinship analysis clustered the sorghum accessions in to two distinct groups in agreement with the results of STRUCTURE (**Figure 3**).

**Figure 4:**
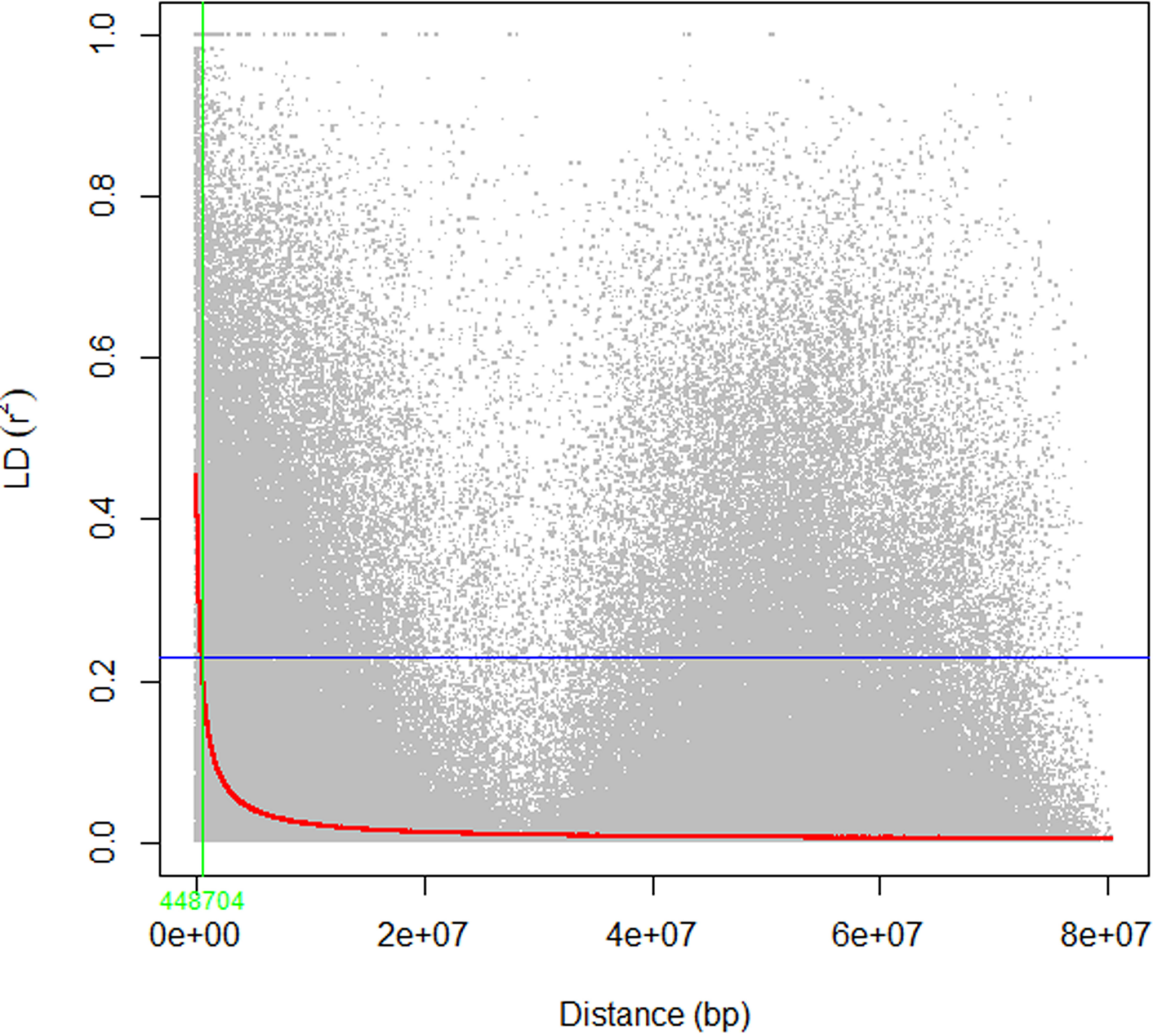
The scatter plot of genome-wide linkage disequilibrium (LD) decay determined based on the r^2^ values of the marker pairs. The red curve line is the smoothing spline regression model fitted to LD decay. The horizontal blue line is the half decay r^2^ value of the genome (r^2^ = 0.23), whereas the vertical green line is the genetic distance between markers in bp (448 kb) at the intersection between the half decay and the LD decay curve.

The mean linkage disequilibrium for the ten chromosomes is quite similar, with an r^2^ value ranging from 0.09 to 0.12 with an overall average of 0.11 (**Table 2**). LD was significant, on average, for 50% (71,611) of the marker pairs (mean r^2^ = 0.2; p ≤ 0.01) (**Table 2**). On average, 35% (24,798) of the significant pairs were physically linked (r^2^ > 0.2) having an r^2^ value 0.42. The highest and lowest number of marker pairs that showed significant LD (p ≤ 0.01) were recorded on Chromosome 3 (8,938; r^2^ = 0.21) and chromosome 7 (5,309; r^2^ = 0.19) (**Table 2**). Among the significant marker pairs, those on chromosome 8 had the strongest LD (mean r^2^ = 0.22), while those on chromosome 5 had the weakest LD (mean r^2^ = 0.18) (**Table 2**). Detailed information regarding the relationship between r^2^ values and physical distances can be found in **Supplementary Table 4**. At the genome level, the r^2^ value was 0.11, and the decay curve of the LD began at r^2^ value of 0.48 and reached half-decay at 0.23. The decay curve of the LD intersected the half-decay line at a distance of 449 kb (**Figure 4**).

**Table 2:** A summary of the results of linkage disequilibrium (LD) analyses among SNP marker pairs, including the number of significant marker pairs for each chromosome.

Chr	Total number of pairs	Average r^2^ for all pairs	D in Mbp	Number of significant pairs*	Average r^2^ for all significant pairs	D in Mbp	Number of PLP(r^2^>0.2)**	D in Mbp	Average r^2^ for PLP	Complete LD (r^2^ = 1)
1	14680	0.11	6.4	7728 (53)	0.20	5.83	2568 (33)	4.80	0.43	59
2	15119	0.11	6.0	7690 (51)	0.21	5.34	2844 (34)	4.42	0.42	72
3	17967	0.11	5.1	8938 (50)	0.21	4.63	3072 (34)	4.04	0.42	64
4	14675	0.11	5.7	7425 (51)	0.2	5.95	2630 (35)	6.96	0.41	68
5	14370	0.09	6.1	6579 (46)	0.18	5.68	2120 (32)	5.00	0.39	43
6	13465	0.11	5.1	6897 (51)	0.21	5.13	2465 (36)	4.91	0.43	80
7	11131	0.10	7.1	5309 (48)	0.19	6.81	1726 (33)	6.96	0.4	59
8	12072	0.12	6.3	6121 (51)	0.22	7.44	2281 (45)	9.20	0.44	52
9	14039	0.11	5.1	7022 (50)	0.21	5.04	2388 (34)	4.60	0.44	102
10	15721	0.11	4.8	7902 (50)	0.2	4.82	2704 (34)	4.50	0.42	49
T/M	143239	0.11	5.8	71611 (50)	0.20	5.67	24798 (35)	5.54	0.42	648

Chr, Chromosome; D, Physical distance, Mbp, Mega base pairs; PLP, Physically linked pairs; T/M, Total/Mean; Significant marker pairs, P < 0.01; * Numbers in the brackets are the percentage of the total number of marker-pairs; ** Numbers in the brackets are the percentage of the number of significant marker pairs.

### 3.3 Marker-trait association for the six target traits

**Figure 5:**
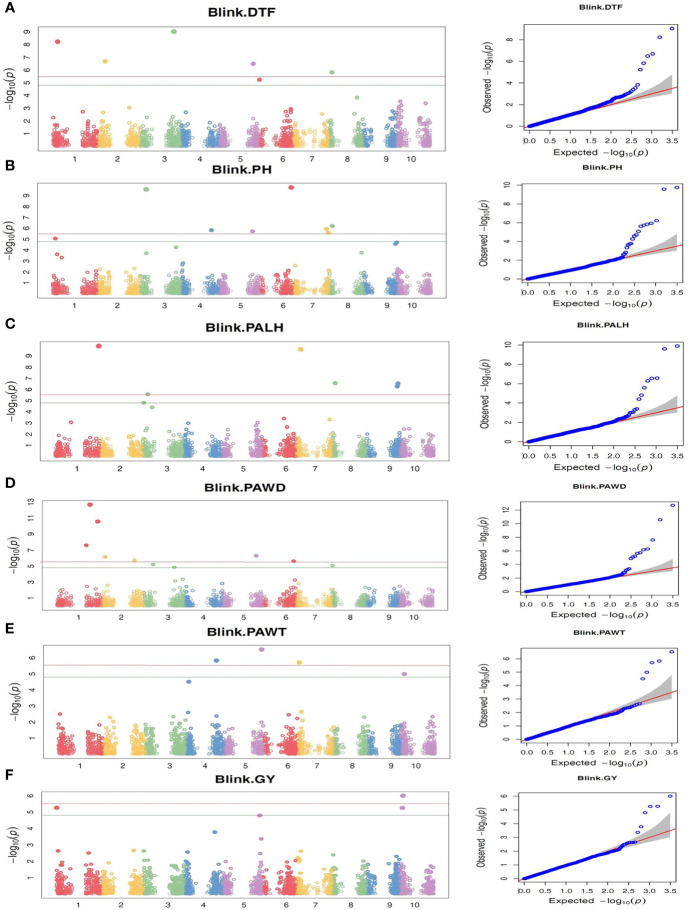
The Manhattan and QQ plots from BLINK-based GWAS analyses of 309 Ethiopian sorghum accessions using 3143 genome-wide SNP markers, revealing markers with significant association (P-value threshold of 0.05 with Bonferroni correction) with the traits targeted in this study. **(A)** DTF, Days to flowering; **(B)** PH, Plant height; **(C)** PALH, Panicle length, **(D)** PAWD, Panicle width, **(E)** PAWT, Panicle weight, and **(F)** GY, Grain yield. The horizontal red and green lines represent P-value threshold of 0.01 and 0.05 with Bonferroni correction, respectively.

The marker-trait association analysis using genome-wide SNP markers was performed using two different multi-locus models, BLINK and farmCPU, which identified 39 and 26 SNP loci with significant association with one or two of the six traits studied, respectively. Among the significantly associated SNP loci, 11 were identified by both models (**Table 3** **)**. Furthermore, three of the SNP loci were associated with two different traits, which means that a total of 51 SNP loci (**Table 3**) were associated with one or more traits. The Manhattan plots, which graphically display the GWAS outputs are provided in **Figure 5** and **Supplementary Figure 3**. The corresponding QQ plots showed that the observed and expected P-values for the vast majority of SNPs are matching, with a clear deviation of the observed values from the expected close to the right end of the plot, suggesting a realistic positive association between the SNPs and the traits **(** **Figure 5** and **Supplementary Figure 3** **).** Hence, the GWAS results are reliable and false negative results are less likely. The map positions of the SNPs associated with the traits, in *Sorghum bicolor* reference genome v3.1.1, enabled the identification of genes harboring those SNPs. The characteristics of those SNPs and the effects they have on their genes were presented in **Supplementary Table 5**.

**Table 3:** List of significant markers associated with agronomic traits from genome-wide association analysis of 309 sorghum accessions using 3143 SNPs with MAF greater than 0.05.

Trait	SNP	Alleles	Chr	Position	MAF	P.value	FDR	PVE (%)	Model
DTF	sbi318688	G/**A**	1	8066218	0.26	4.14E-06	0.002842	2.26	farmCPU
						5.89E-09	9.26E-06	1.45	BLINK
	sbi982537	G/**T**	1	22275540	0.12	1.03E-05	0.004032	2.38	farmCPU
	sbi4023260	G/**C**	2	13293723	0.43	2.07E-07	0.000217	2.09	BLINK
	sbi10226120	A/**G**	3	56322246	0.29	9.58E-06	0.004032	3.58	farmCPU
	sbi10372954	T/**C**	3	59472033	0.13	2.24E-06	0.002345	0.91	farmCPU
	sbi10438246	**C**/A	3	60811994	0.06	7.88E-11	2.48E-07	23.95	farmCPU
						9.32E-10	2.93E-06	35.23	BLINK
	sbi17364528	T/**G**	5	61608548	0.07	3.24E-07	0.000255	9.89	BLINK
	sbi17966906	C/**A**	6	1001235	0.29	5.84E-06	0.003064	1.83	BLINK
	snp_sb001000704585	A/**T**	6	48682625	0.32	9.37E-06	0.004032	1.00	farmCPU
	snp_sb042060813825	A/**G**	7	39653062	0.07	2.05E-06	0.002345	2.59	farmCPU
	**sbi24678469** ^b^	**C**/G	8	5242533	0.09	1.51E-06	0.00095	3.97	BLINK
	sbi30754782	**G**/T	10	9494280	0.26	4.52E-06	0.002842	1.96	farmCPU
PH	sbi62970	**C**/T	1	1495213	0.11	1.45E-05	0.0046	4.35	farmCPU
	sbi194467	G/**A**	1	4778713	0.40	8.41E-06	0.0033	0.71	BLINK
	sbi7769289	C/**T**	3	10836535	0.08	1.20E-05	0.004207	3.11	farmCPU
						2.70E-10	4.25E-07	6.32	BLINK
	sbi7901589	A/**G**	3	13494213	0.38	1.85E-06	0.0012	6.32	farmCPU
	**sbi13732034** ^a^	C/**T**	4	54108806	0.19	1.91E-07	0.000201	2.21	farmCPU
						1.47E-06	0.000923	3.21	BLINK
	sbi17260778	**G**/A	5	59844243	0.42	1.89E-06	0.0010	2.46	BLINK
	sbi20359577	**C**/T	6	48007508	0.13	3.56E-06	0.0016	1.75	farmCPU
	sbi20792384	**G**/T	6	57704110	0.31	1.29E-12	4.05E-09	1.62	farmCPU
						1.77E-10	4.25E-07	1.43	BLINK
	sbi24138103	**G**/T	7	59997937	0.12	1.15E-06	0.0009	1.59	BLINK
	sbi24291993	T/**C**	7	63300346	0.13	2.46E-06	0.0011	4.58	BLINK
	sbi24668980	**G**/A	8	5053406	0.09	2.56E-06	0.001343	3.77	farmCPU
						5.97E-07	0.000627	1.28	BLINK
	sbi30188088	**C**/G	9	56601353	0.19	2.76E-07	0.0002	0.79	farmCPU
	sbi30546176	C/**T**	10	5270775	0.11	7.45E-06	0.0029	1.55	farmCPU
	sbi30645260	A/**G**	10	7303004	0.06	4.02E-08	0.0001	6.75	farmCPU
PALH	sbi3208134	**C**/T	1	78277352	0.09	2.26E-09	7.10E-06	5.44	farmCPU
						1.30E-10	4.01E-07	9.62	BLINK
	sbi7353016	**G**/A	3	1905405	0.10	1.54E-05	0.006929	4.03	BLINK
	sbi7686697	**A**/G	3	9057564	0.42	2.69E-06	0.00141	2.47	BLINK
	sbi21359653	A/**G**	7	8646573	0.36	2.55E-10	4.01E-07	11.25	BLINK
	**sbi24678469** ^b^	C/**G**	8	5242533	0.09	2.67E-07	0.000228	3.24	BLINK
	sbi30118348	T/**A**	9	54963433	0.34	5.18E-07	0.000326	1.74	BLINK
	sbi30169733	**A**/T	9	56240567	0.26	1.41E-06	0.002214	2.87	farmCPU
						2.89E-07	0.000228	18.91	BLINK
PAWD	sbi2029574	G/**T**	1	52905292	0.20	6.67E-06	0.005249	4.80	farmCPU
						2.43E-08	2.55E-05	0.44	BLINK
	sbi2393610	**A**/T	1	59685990	0.07	9.96E-08	0.000313	10.42	farmCPU
						2.01E-13	6.33E-10	23.31	BLINK
	sbi3026667	T/**C**	1	73774042	0.21	2.59E-11	0.005249	4.51	BLINK
	sbi3655489	A/**T**	2	6615133	0.30	6.97E-07	0.002386	1.45	BLINK
	sbi6496334	G/**T**	2	59698705	0.22	1.90E-06	0.002902	1.34	BLINK
	sbi8085609	C/**G**	3	16868775	0.07	5.49E-06	0.005249	2.19	farmCPU
						6.07E-06	0.002386	10.93	BLINK
	sbi10162413	T/**C**	3	55078201	0.25	1.35E-05	0.000438	0.94	BLINK
	sbi10836055	T/**C**	3	70604368	0.19	4.19E-06	2.55E-05	6.37	farmCPU
	sbi17331791	G/**C**	5	61061450	0.21	5.14E-07	0.004239	1.57	BLINK
	sbi20777395	A/**G**	6	57341190	0.18	2.29E-06	6.33E-10	2.05	BLINK
	sbi24445033	G/**T**	8	1016470	0.27	8.30E-06	4.08E-08	2.23	BLINK
PAWT	**sbi13732034^a^**	C/**T**	4	54108806	0.19	1.46E-06	0.002048	5.91	BLINK
	sbi17687423	**T**/C	5	67739573	0.08	3.04E-07	0.000956	7.01	BLINK
	sbi21079990	**A**/G	7	2945057	0.36	1.95E-06	0.002048	1.65	BLINK
	sbi21265823	T/**C**	7	6671420	0.30	1.50E-05	0.026887	8.03	farmCPU
	**sbi30583046^c^**	**C**/G	10	6047480	0.50	1.01E-05	0.00798	2.69	BLINK
GY	sbi3833	T/**C**	1	80269	0.2	5.43E-06	0.005837	3.10	BLINK
	sbi17653919	G/**C**	5	67092426	0.1	1.59E-05	0.012496	3.12	BLINK
	sbi17789352	G/**A**	5	69674648	0.2	3.13E-06	0.009845	11.49	farmCPU
	snp_sb001001045636	A/**G**	10	5151950	0.1	5.57E-06	0.005837	6.69	BLINK
	**sbi30583046^c^**	C/**G**	10	6047480	0.5	1.00E-06	0.003155	7.23	BLINK

MAF, Minor allele frequency; Chr, chromosome; PVE, Phenotypic variance explained; FDR, False discovery rate; PH, Plant height; DTF, Days to flowering; PALH, Panicle length; PAWD, Panicle width; PAWT, Panicle weight; GY, Grain yield. Favorable alleles are given in bold. SNP associated with more than one trait are shown in bold, and those sharing a superscript are the same.

#### 3.3.1 Days to flowering (DTF)

**Figure 6:**
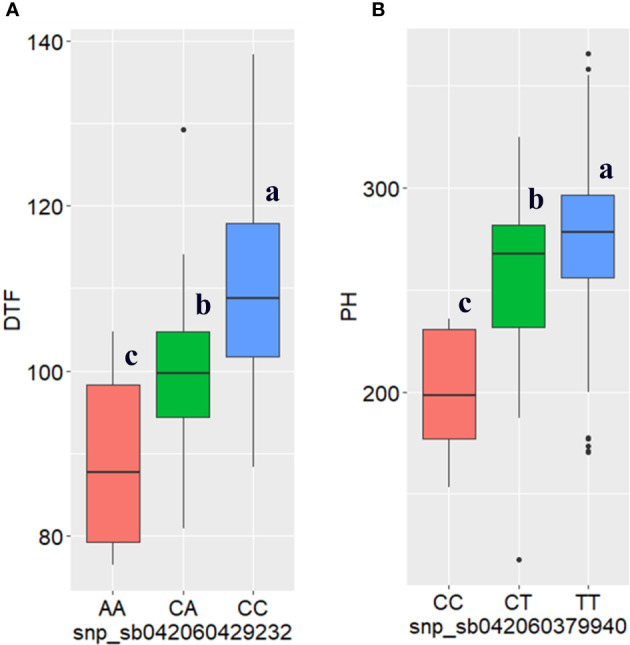
Boxplots of high signal SNPs (sbi10438246 and sbi7769289) showing the effects of their alleles on **(A)** days to flowering (DTF) and **(B)** plant height (PH), respectively, generated based on the best linear unbiased predictor analysis of the combined phenotypic data from the three locations. Statistical significance for differences between alleles in the 309 sorghum genotypes was determined using the Tukey’s HSD (honestly significant difference) test. The different letters in the box plot indicates the significance difference among the mean P < 5% level.

In the genome-wide association study, a total of 12 SNPs with significant associations with days to flowering were identified, through either the BLINK model or farmCPU model, or both (**Table 3** **)**. These markers are distributed across all chromosomes, except chromosomes 4 and 9. Two of the markers were identified through both BLINK and farmCPU models on chromosome 1 (sbi318688; position 8066218 bp) and chromosome 3 (sbi10438246; position 60811994) (**Table 3**; **Figure 5**, and **Supplementary Figure 3** **)**. The phenotypic variance of the trait explained by the significant SNPs ranged from 0.9 to 35% (**Table 3**). The marker sbi10438246 on chromosome 3, identified by both models, accounted for the highest percentage of the variance in DTF (35%; the strongest MTA signal) (**Table 3**). Box plots depicting the effects of the alleles at this locus for this trait are shown in **Figure 6A**. The SNP, a missense variant that alters serine versus tyrosine, is located within the coding sequence (CDS) of the gene Sobic.003G271700, which codes for a protein of unknown function.

The second major effect marker (a missense mutation on chromosome 5), accounting for 9.9% of the DTF variance, is located within gene Sobic.005G147700, which codes for extensin-2-like protein (**Table 3** and **Supplementary Table 5**). The SNP sbi24678469 and sbi982537 are DTF-associated markers present within the coding sequences of the genes Sobic.008G052000 and Sobic.001G230700, respectively. These genes encode fatty acid amide hydrolase, and RING finger and E3 ubiquitin-protein ligase MIEL1. For Sobic.001G230700 the most significant hit (homologue) in rice is RING finger and CHY zinc finger domain-containing protein 1, which has been shown to be involved in the regulation of seedling development and flowering time. Sobic.007G109800 (containing snp_sb042060813825) encodes late embryogenesis abundant protein D-34 (a seed maturation protein), which is involved in seed development and maturation, as well as response to biotic stress. Other genes containing the DTF-associated SNP markers include white-brown complex homolog protein 11, ATP binding microtubule motor family protein, phototropic-responsive NPH3 family protein and tetratricopeptide repeat (TPR)-like superfamily protein, and phototropin 1 (phot1) (**Supplementary Table 5**).

#### 3.3.2 Plant height (PH)

In this study, the highest number of significant MTA was identified for plant height. In total, 14 SNPs with significant association with plant height were identified by BLINK and farmCPU models (**Table 3**, **Figure 5** and **Supplementary Figure 3** **)**. Both BLINK and farmCPU identified four significant markers on chromosomes 3, 4, 6, and 8. The percentage of phenotypic variance explained by these markers ranged from 1.6% (on chromosome 6) to 6.3% (on chromosome 3) (**Table 3**). The effects of alleles on PH at sbi7769289 locus that accounted for the highest phenotypic variance (6.5%) and highest MTA signal are shown with box plots (**Figure 6B**). Additionally, four markers were identified only by BLINK on chromosomes 1, 5, and 7, explaining phenotypic variance ranging from 0.7 (chromosome 1) to 4.6% (chromosome 7) (**Table 3** and **Figure 5** **)**. Similarly, six significant markers, on chromosomes 1, 3, 6, 9, and 10 were identified only by the farmCPU model, explaining phenotypic variance ranging from 0.8 (on chromosome 9) to 6.8% (on chromosome 10) (**Table 3** and **Supplementary Figure 3** **)**.

All markers with significant association with PH were located within genes (**Supplementary Table 5**). The two genic SNP markers that had the largest effects on PH, on chromosome 3 at 11 Mb (PVE = 6.3%) and 10 at 7 Mb (PVE = 6.8%) (**Table 3**) were located within the genes, Sobic.003G119600 and Sobic.010G085400, respectively. Sobic.003G119600 encodes zinc finger and C3HC4 type domain- containing protein while Sobic.010G085400 encodes K-box region and MADS-box transcription factor family protein. These genes play a significant role in various physiological and cellular processes including transcription, signal transduction, recombination, plant growth, and vegetative and reproductive developments (**Supplementary Table 5**). Sobic.001G017500 (encoding hydroxysteroid dehydrogenase), and Sobic.009G223500 (encoding F-box family protein), which are known for their role in plant vegetative and reproductive growth and development, contain significant SNPs associated with PH. Similarly, Sobic.006G111800 (encoding ARM repeat superfamily protein), Sobic.006G235400 (encoding protein kinase superfamily protein), and Sobic.010G066100 (encoding phototropic-responsive NPH3 family protein) also had significant markers associated with PH. These genes are involved in the regulation of growth and development, stress signaling, and phototropin 1 signaling (**Supplementary Table 5**).

#### 3.3.3 Panicle length (PALH)

The GWAS identified seven SNPs with significant association to PALH across five different chromosomes (1, 3, 7, 8 and 9) (**Table 3**; **Figure 5**, and **Supplementary Figure 3** **)**, and all of them are located within genes (**Supplementary Table 5**). Two of these SNP markers were identified by both farmCPU and BLINK models, and explained 9.6% (sbi3208134; on chromosome 1) and 18.9% (sbi30169733; on chromosome 1) of the phenotypic variance of PALH (**Table 3**). The sbi3208134 is located within Sobic.001G516100, which encodes BR-signaling kinase 1 while sbi30169733 is located within Sobic.009G218450, which encodes P-glycoprotein 11, MDR-like ABC transporter (**Table 3** and **Supplementary Table 5**). The other five markers (located on chromosomes 3, 7, 8 and 9) were detected only by BLINK model, and accounted for up to 11.3% of the total phenotypic variance of the trait. The genes harboring these SNPs encode for proteins of different functions (**Supplementary Table 5**). Among these genes, Sobic.009G199900 (on chromosome 9) contains the sbi30118348, which encodes phosphatidylethanolamine binding protein (PEBP), which is an important factor in regulating flowering in response to photoperiod.

#### 3.3.4 Panicle width (PAWD)

The GWAS analyses detected eleven SNPs with significant association with PAWD (**Table 3**; **Figure 5** and **Supplementary Figure 3** **)**. Three of these SNPs: sbi2029574 and sbi2393610 (on chromosome 1), and sbi8085609 (on chromosome 3) were detected by both BLINK and farmCPU. Among them, sbi2393610, which accounted for 23.3% of the variation in PAWD, is located within the Sobic.001G310300 gene that encodes glutathione S-transferase F11. Whereas, sbi8085609, which accounted for 10.9% of the variation in PAWD, is located in Sobic.003G154800 that encodes protein of unknown function (DUF594) (**Table 3** and **Supplementary Table 5**).

Among the remaining eight markers, seven (on chromosomes 1, 2, 3, 5, 6 and 8) were detected only by BLINK and one (on chromosome 3) was detected only by farmCPU. The phenotypic variance of the trait explained by these SNPs ranged from 0.9 to 6.4% (**Table 3**). These include SNPs within genes encoding appr-1-p processing enzyme family protein (Sobic.003G215900), RNA-binding family protein (Sobic.005G145400), S-locus lectin protein kinase family protein (Sobic.006G229100), protein kinase family protein with leucine-rich repeat domain (Sobic.001G273500), and magnesium transporter 4 (Sobic.003G395600) (**Supplementary Table 5**).

#### 3.3.5 Panicle weight (PAWT)

The GWAS analyses detected five SNPs that were significantly associated with PAWT, explaining 1.7 to 8.0% of its phenotypic variance (**Table 3**, **Figure 5** and **Supplementary Figure 3** **)**. Four of these markers (on chromosomes 4, 5, 7 and 10) were identified by BLINK model whereas one marker (on chromosome 7) was detected by farmCPU model. The SNP marker sbi21265823 (on chromosome 7), which accounted for 8.0% of the PAWT phenotypic variance, is located within the Sobic.007G033500 gene, which encodes a protein of unknown function (**Supplementary Table 5**). Among the remaining four markers, sbi17687423 (on chromosome 5), which accounted for 7% of the phenotypic variance of PAWT, is located within the Sobic.005G194000 gene, which encodes for a protein of unknown function. Whereas, sbi13732034 (on chromosome 4), which explained 5.9% of the phenotypic variance of PAWT, is located within the Sobic.004G189200 gene, which encodes for F-box domain and kelch repeat containing protein (**Table 3** and **Supplementary Table 5**).

#### 3.3.6 Grain yield (GY)

GWAS identified five SNPs significantly associated with GY (**Table 3**, **Figure 5** and **Supplementary Figure 3** **)**. BLINK model identified four of these markers (on chromosomes 1, 5 and 10) whereas farmCPU model identified one marker (on chromosome 5). The percentage of phenotypic variance explained by these markers ranged from 3.1% (sbi3833 and sbi17653919; on chromosomes 1 and 5, respectively) to 11.5% (sbi17789352 on chromosome 5). The SNP marker sbi17789352 is located within the Sobic.005G209900 gene, which codes for protein of unknown function. The two SNP markers on chromosome 10, explained 6.7% (snp_sb001001045636) and 7.2% (sbi30583046) of the variation in grain yield. The SNP sbi30583046 is located within the coding sequence of the Sobic.010G074100 gene. This gene codes for a pentatricopeptide repeat (PPR) superfamily protein that plays a role in physiological processes contributing to plant growth and development (**Table 3** and **Supplementary Table 5**).

## 4 Discussion

### 4.1 Phenotypic variation

Genome-wide association mapping is a powerful method that facilitates the eventual identification of genes regulating traits of interest, whose efficiency are dependent on the genetic variation within the germplasm used as association panels. In this study, high phenotypic variation was observed within the Ethiopian sorghum accessions used as GWAS panel (as revealed by descriptive statistics) for each of the six target traits. Similarly high phenotypic variation was reported in previous studies on Ethiopian sorghum landrace accessions (Girma et al., 2019; Enyew et al., 2021). Hence, the sorghum landraces used for this study had a sufficiently high phenotypic variation that makes them suitable for use as GWAS panel as well as for selecting genotypes with desirable traits for use in sorghum breeding programs.

The influence of environments and G×E were lower on DTF, PH, PALH, and PAWD, indicating a predominant contribution of genotypic variation to the high phenotypic variation in these traits. On the other hand, the contribution of genotypic variation to the phenotypic variation of PAWT and GY was low. For these traits, the vast majority of the phenotypic variation was due to the variances of environment and G×E, of which environment was a dominant factor. This was shown by a significantly lower broad-sense heritability of PAWT and GY as compared to that of DTF, PH, PALH, and PAWD. The high heritability for DTF, PH, and PALH and moderate heritability for GY were also reported in previous studies on sorghum (Amare et al., 2015; Zhao et al., 2016; Habyarimana et al., 2020; Luo et al., 2020). Overall, the large variation of the evaluated sorghum accessions and moderate to high broad-sense heritability of the traits suggest the significance of these genetic resources both for crop improvement through breeding as well as for the identification of genes regulating these traits.

### 4.2 Linkage disequilibrium

Identifying the pattern of LD is crucial to design association studies and molecular breeding strategies (Thornsberry and Buckler, 2003). To characterize the LD decay in this study, LD was calculated at chromosome and genome levels using the SNP data from the 309 sorghum accessions. In this study, the average r^2^ value was 0.11 at the genome level. The LD started decaying at r^2^ value of 0.48 and reached its half-decay value (r^2^ = 0.23) by 448 kb. The LD decay curve intersected with the half-decay at a distance of 448 kb. These LD decay estimates are similar to the previously published value within 500 kb in sorghum (Marla et al., 2019). However, the estimates are higher than previously published values of r^2^ < 0.1 within 150 kb (Morris et al., 2013) and 100 kb (Bouchet et al., 2012). The average r^2^ in each sorghum chromosomes have similar rate of LD decay, between 0.09 and 0.12 which is in agreement with previous studies on sorghum (Wang et al., 2013; Hu et al., 2019).

### 4.3 Genome-wide associations and candidate gene identifications for agronomic traits

In this study, two multi-locus GWAS models were used for GWAS to overcome the limitations arising from using single-locus models (Liu et al., 2016; Li et al., 2018a). The multi-locus models avoided the confounding effects of population structure by including kinship and principal components in the GWAS models. The QQ plots also confirmed that the power of the models to detect true marker-trait associations was high. In total, 51 MTAs were identified in this study with the number of MTAs for each trait ranging from five (for PAWT and GY) to 14 SNPs (for PH). Among the significant SNPs, 11% explained over 10% of the phenotypic variation of the corresponding traits. Whereas, 31% of the SNPs explained over 5% of the phenotypic variation of the corresponding traits. The significant markers together accounted for 25.3% (for PAWT) to 67.7% (for DTF). Therefore, sorghum could be improved significantly by pyramiding favorable alleles using the marker-assisted selection (MAS) approach using the MTAs identified for each trait.

#### 4.3.1 Days to flowering

The Maturity (Ma) loci (Ma1-Ma6) have been shown to regulate sorghum flowering time (Quinby and Karper, 1953; Rooney and Aydin, 1999). The Ma1 locus represent the Sobic.006G057866 gene located on chromosome 6, which encodes pseudo-response regulator protein 37 (PRR37). This gene has a significant effect on flowering time by controlling floral inhibitors and activators (Murphy et al., 2011). Ma2 represents the Sobic.002G302700 gene located on chromosome 2, which encodes a SET and MYND (SYMD) domain-containing lysine methyl transferase (Casto et al., 2019). Both Ma3 and Ma5 are located on chromosome 1 and represent the Sobic.001G394400 and Sobic.001G087100 genes, respectively. These genes encode phytochrome B (Childs et al., 1997) and phytochrome C (Yang et al., 2014), respectively. Ma6 represents Sobic.006G004400 gene located on chromosome 6, which encodes Ghd7, a CONSTANS, CO-like, and TOC1 (CCT)-domain protein (Murphy et al., 2014). The gene for the Ma4 locus has not yet been identified.

Among 12 SNP loci identified as being associated with DTF in this study, seven are located in close proximity with previously identified marker loci for the same trait (**Supplementary Table 6**). For example, the sbi982537 marker on chromosome 1 (at position 22.3 Mb) is in close proximity with the previously reported marker loci located at 21.5 Mb (Ritter et al., 2008) and 23.1 Mb (Kong et al., 2018), suggesting that they might refer to the same QTL. As sbi982537 is located within the CDS (causing missense mutation) of Sobic.001G230700, which codes for RING finger and CHY zinc finger domain-containing protein 1, it could be a potential candidate gene behind the QTL these markers share. The map position of sbi982537, however, is 15.5 Mb and 45.7 Mb away from Ma3 and Ma5 loci, respectively, making it unlikely that they are associated. In contrast, sbi318688 is located only 1.3 Mb from a well characterized maturity loci Ma5 (Sobic.001G087100), at position 6.7 Mb on chromosome-1 (Yang et al., 2014), which suggests that they may be associated. There is also a possibility that the Sobic.001G105400 gene containing sbi318688 is responsible for the phenotypic variation since its orthologue plays an important role in pollen development and seed set in rice (Zhang et al., 2020).

In this study, three SNP loci located close to one another on chromosome 3 (56.3 Mb to 60.8 Mb) were found to be associated with DTF, and it is possible that they refer to the earlier identified QTL (Kong et al., 2018). This QTL region contains the sbi10438246 (located at position 60.8 Mb) that explained over a third (35.2%) of the phenotypic variation in DTF. This SNP is a missense mutation within the Sobic.003G271700 gene that encodes a protein of unknown function. It is therefore imperative to conduct further research to determine whether this gene or another gene nearby is responsible for the phenotypic variation the SNP explained. The SNP snp_sb001000704585 locus (a missense mutation) on chromosome 6 explaining 1% of the variation in DTF is located within the Sobic.006G120000 gene that encodes the phototropic-responsive NPH3 family protein, which is known to play important roles in photo-signaling in addition to phototropism (Christie et al., 2018), suggesting that it may be causal. Similarly, the sbi24678469 locus located at 52.4 Mb on chromosome 8 was co-localized with the previously identified QTLs (Zhao et al., 2016; Kong et al., 2018).

The snp_sb042060813825 locus on chromosome 7 at position 39.7 Mb represents a novel QTL for DTF, as no MTA for this trait has been identified in this genomic region in previous studies. This SNP locus is located within the Sobic.007G109800 gene, which encodes for a protein referred to as seed maturation protein or late embryogenesis abundant protein D-34. The gene is involved in the regulation of seed maturation (Hundertmark and Hincha, 2008), and is probably behind the variation in DTF explained by this SNP locus. Another SNP locus, sbi17364528, at 61.6 Mb on chromosome 5 was located about 10 Mb away from the previously identified locus for DTF (Zhao et al., 2016). This SNP locus (a missense mutation) is located within the Sobic.005G147700 gene, which encodes a protein of unknown function. As the SNP explained 9.9% of the variation in DTF, it represents a major QTL, while the gene is likely a novel locus that regulates flowering time in sorghum. Similarly, an SNP locus at 13.3 Mb on chromosome 2 was located about 54 Mb away from the previously identified well-known maturity locus (Ma2) (Zhao et al., 2016).

#### 4.3.2 Plant height

Sorghum possesses four major genomic loci that control plant height, known as dwarfing loci (Dw1-Dw4) (Quinby and Karper, 1953), of which Dw1, Dw2, and Dw3 have been characterized. Dw1 encodes a putative membrane protein (Sobic.009G230800), which has a role in controlling cell proliferation of internodes (Hilley et al., 2016; Yamaguchi et al., 2016). Dw2 encodes a protein kinase (Sobic.006G067600), which regulates the length of stem internodes  (Hilley et al., 2017). Dw3, which encodes a phosphoglycoprotein of the adenosine triphosphate-binding cassette (ABC) transporter superfamily (Sobic.007G163800), plays an important role in auxin transport (Multani et al., 2003). Dw4 has been mapped to chromosome 4 (Li et al., 2015), but the gene behind this locus has not been identified yet. Additionally, a fifth dwarfing locus (Dw5) has recently been reported (Chen et al., 2019).

In this study, an SNP locus (sbi30188088) at 56.6 Mb on chromosome 9 showed a significant association with plant height. This locus is located only ca 3 Mb from the major dwarfing locus, Dw1, which lies at 59.6 Mb (Hilley et al., 2016; Yamaguchi et al., 2016). Nevertheless, it is unlikely that this SNP refers to Dw1, considering its minor effect on the variation in PH. Two recent GWAS on sorghum identified SNPs associated with plant height at 56.6 and 56.5 Mb (Habyarimana et al., 2020; Luo et al., 2020), which are co-localized with sbi30188088. Moreover, sbi30188088 is located within the Sobic.009G223500 gene, which encodes an F-box family protein that has known role in ethylene and gibberellic acid signaling (Dill et al., 2004) as well as acting as auxin receptors in Arabidopsis to regulate the stability of indole 3-acetic acid (IAA) proteins (Kepinski and Leyser, 2005). Therefore, it is likely that Sobic.009G223500 is a gene behind the PH variation explained by sbi30188088.

Several linkage mapping and GWAS identified loci associated with plant height on chromosome 6 between 42.2 to 61.5 Mb (Wang et al., 2012; Morris et al., 2013; Burks et al., 2015; Zhang et al., 2015; Kong et al., 2018; Habyarimana et al., 2020). In the present study, two SNP loci located at positions 48.0 Mb and 57.7 Mb were identified in this genomic region although their effect on the phenotype was low (below 2%). Considering their low effect and large distance (6-15 Mb) from the Dw2 locus located at 42.2 Mb on this chromosome, which encodes a protein kinase that regulates stem internode length (Hilley et al., 2017), it is unlikely that the SNPs refer to Dw2. Nevertheless, these SNPs are the result of missense mutations in the Sobic.006G111800 and Sobic.006G235400 genes, which encode ARM repeat and protein kinase, respectively. It is possible that these mutations are causal for the variation in PH associated with these SNPs. The sb042060843522 locus associated with PH is within Sobic.007G165200 gene on chromosome 7 (at 60.0 Mb), which is only about 1.6 Mb away from the Dw3 gene. It is also co-localized with the previously identified QTL on chromosome 7 at 59.6 Mb in the Ethiopian sorghum landrace (Girma et al., 2019).

The SNP locus for PH, sbi13732034, which is located at 54.1 Mb on chromosome 4, is in close proximity with a PH associated locus (at 52.6 Mb) reported by (Zhang et al., 2015). Although the exact location and the gene behind it are yet to be confirmed, the Dw4 locus has been mapped close to the end of chromosome 4 (Li et al., 2015). It is unlikely that sbi13732034 refers to Dw4, as its impact on PH variation is quite small (3.2%), and their map positions are quite different. However, sbi13732034 is located within the Sobic.004G189200 gene, which encodes F-box domain and kelch repeat containing protein. As discussed above, this protein has a known role in ethylene and gibberellic acid signaling (Dill et al., 2004) and auxin receptors in Arabidopsis (Kepinski and Leyser, 2005), and it is possible that it is an underlying gene for the variation explained by sbi13732034.

Two SNP loci (sbi7769289 and sbi7901589) located at positions 10.8 and 13.5 Mb on chromosome 3 were found to be associated with PH in this study. None of previously identified loci for PH was mapped to this genomic region. Considering the fact that the map distance between the two SNPs is 2.7 Mb, they may refer to the same gene, as both explained 6.2% of the observed phenotypic variation. The SNPs are located within the genes Sobic.003G119600 and Sobic.003G139200, respectively. Sobic.003G119600 encodes RING/U-box superfamily protein, zinc finger, C3HC4 type domain containing protein which is involved in plant growth and development (Wu et al., 2014), and it is possible that it is the gene behind the variation observed. Another novel major effect SNP locus (sbi30645260) located within the Sobic.010G085400 gene (at 7.3 Mb) on chromosome 10 was also identified for PH. The gene ontology (GO) analysis of this gene revealed that it is associated with four GO terms. In the molecular function (MF) GO class, it is annotated as RNA polymerase II cis-regulatory region sequence-specific DNA binding (GO:0000978), and DNA-binding transcription factor activity, RNA polymerase II-specific (GO:0000981_MF). Under biological process (BP) GO class, it is annotated as regulation of transcription by RNA polymerase II (GO:0006357), and transcription by RNA polymerase II (GO:0006366). This candidate gene encodes MADS-box transcription factor family protein, which is known to be involved in the regulation of flowering time in Arabidopsis (Michaels and Amasino, 1999), vegetative and root growth (Zhang et al., 2018), as well as other functions, such as floral organ development (Dreni and Zhang, 2016). Further study may shed light as to whether it is involved in the regulation of plant height in sorghum.

#### 4.3.3 Inflorescence architecture

In this study, significant SNP loci for panicle length were identified on chromosomes 1, 3, 7, 8 and 9. Previous linkage mapping and GWAS in sorghum detected MTA for panicle length on all chromosomes (**Supplementary Table 6**). This study identified three major effect SNP loci (sbi3208134, sbi21359653, and sbi30169733) on chromosome 1 at 78.2 Mb (PVE = 9.7%), chromosome 7 at 8.6 Mb (PVE = 11.2%), and chromosome 9 at 56.2 Mb (PVE = 18.9%) that showed strong association signals for panicle length. These SNPs are located within the genes Sobic.001G516100, Sobic.007G075200, and Sobic.009G218450, respectively. They encode brassinosteroid (BR) signaling kinase 1, bifunctional purine biosynthesis protein, and P-glycoprotein 11, MDR-like ABC transporter, respectively. The BR signaling pathway is known to play a role in plant cell elongation and division, tissue differentiation, organogenesis (Sakamoto et al., 2006), and inflorescence architecture (Li et al., 2018b). In order to determine if any of these genes are directly involved in the regulation of panicle length in sorghum, further research is required. Two SNP loci at 1.9 and 9.0 Mb on chromosome 3 were located in close proximity with previously reported loci at 1.8 Mb (Habyarimana et al., 2020) and 9.9 Mb (Zhao et al., 2016), respectively. On chromosome 9, two SNP loci at 55.0 and 56.2 Mb were found in close proximity with previously reported SNP locus at 55.3 Mb (Habyarimana et al., 2020).

Six of the eight SNP loci with significant association with panicle width (PAWD) were located in close proximity with the previously reported loci associated with this trait in sorghum (**Supplementary Table 5**). For instance, two major effect SNPs on chromosome 1 at 59.7 and 73.7 Mb were co-localized with previously reported SNP loci for PWAD in sorghum (Zhang et al., 2015). The sbi2393610 at 59.7 Mb on chromosome 1 was located about 1.4 Mb away from the previously identified locus for PAWD (Zhang et al., 2015). This SNP is located within the gene Sobic.001G310300, which encodes glutathione S-transferase F11. Three GO terms were associated with this gene under the molecular function (MF) gene ontology class. These are glutathione transferase activity (GO:0004364), ion binding (GO:0043168), and amide binding (GO:0042277). In the cellular component (CC) GO class, the gene is associated with intracellular anatomical structure (GO:0005737), and it is likely to regulate PAWD. As the SNP explained 23.3% of the variation in PAWD, it represents a major QTL that regulate panicle width in sorghum. Similarly, sbi3026667 at 73.7 Mb (PVE = 4.5) on chromosome 1 was located about 1.3 Mb away from the previously identified locus for PAWD (Zhang et al., 2015).

The sbi2029574 on chromosome 1 at 52.9 Mb (PVE = 4.8) which showed a strong association signal for PAWD represents a novel genomic region associated with this trait. The gene containing this SNP, Sobic.001G273500, encodes a kinase family protein with a leucine-rich repeat domain. Further research will shed light if this gene is involved in regulating PAWD. On chromosome 3, three SNP loci at positions 16.9, 55.1 and 70.6 Mb (PVE = 10.9, 1.0, and 6.4, respectively) were in close proximity with previously reported QTLs for PAWD in sorghum (Zhang et al., 2015). The sbi8085609, at 16.9 Mb on chromosome 3 was located about 4 Mb away from the previously identified locus for PAWD (Zhang et al., 2015). This SNP locus (a missense mutation) is located within the Sobic.003G154800 gene, which encodes a protein of unknown function. As the SNP explained 10.9% of the variation in PAWD, it represents a major QTL, while the gene is likely regulates PAWD in sorghum, which needs further investigation.

#### 4.3.4 Panicle weight and grain yield

Previous linkage mapping and GWAS detected QTLs for panicle weight (PAWT) on chromosomes 1, 4, 6, 7, and 9 (**Supplementary Table 5**). None of them, however, is located close to the SNPs on chromosomes 4, 5, 7, and 10 that were found to be associated with PAWT in the present study. The sbi13732034 locus on chromosome 4, which explained 5.9% of the variation in PAWT, is located within the Sobic.004G189200 gene. This gene encodes a protein containing F-box domains and kelch repeats, which modulates ethylene and gibberellic acid signaling (Dill et al., 2004), regulates leaf senescence, seed size and number, and panicle architecture (Chen et al., 2013). It serves as an auxin receptor (Kepinski and Leyser, 2005) in different plant species. Therefore, Sobic.004G189200 is likely to be one of the major genes regulating panicle weight in sorghum. On chromosome 7, the sbi21265823 at 6.7 Mb was associated with PAWT, which might be considered as novel region controlling the trait. This SNP explained 8.0% of the variation in PAWT and is located within Sobic.007G063500 that encodes a protein of unknown function. It is therefore important to carry out further research to know whether this gene or another gene nearby is responsible for the phenotypic variation the SNP explained.

Previous association mapping studies detected several QTLs for grain yield on all chromosomes except chromosomes 1 and 4 (**Supplementary Table 5**). The five SNPs on chromosomes 1, 5, and 10 that were associated with grain yield (GY) in the present study are distant from the previously reported QTLs for GY in sorghum. Therefore, these SNPs probably represent novel QTLs. The sbi17789352 locus on chromosome 5 at position 69.7 Mb represents a novel QTL for GY since no MTA for this trait has previously been identified in this genomic region. This SNP explained 11.5% of the phenotypic variation in GY and located within the Sobic.003G271700 gene that encodes a protein of unknown function. Nevertheless, further research is required to confirm the association between this genomic region and grain yield, and to determine whether Sobic.003G271700 is the gene responsible for the observed variation associated with the SNP. The sbi30583046 locus at position 60.7 Mb on chromosome 10 explained 2.3% and 7.2% variations in PAWT and GY, respectively, which probably refers to a single gene that regulates both PAWT and GY. The SNP locus (missense mutation) is located within the gene Sobic.010G074100, which encodes pentatricopeptide repeat (PPR) superfamily protein. The protein has been reported to play a role in pollen development and seed setting in rice (Zhang et al., 2020), and pollen and organ development in *Arabidopsis* (Prasad et al., 2005). Thus, it is likely that this SNP locus represents a novel QTL that regulates PAWT and GY in sorghum, which is an interesting finding that deserves further research.

## 5 Conclusion

This study used large number of Ethiopian sorghum accessions and gene-based SNP markers to identify genomic regions and candidate genes associated with grain yield and agronomic traits in sorghum. The population structure analysis revealed two genetic populations representing the sorghum accessions studied indicating the presence of stronger genetic relationships among individuals within each genetic population than the overall average. A number of novel and previously known genomic regions that are associated with the studied traits were identified in this study. It is expected that the identified MTAs will contribute significantly to the existing knowledge base of sorghum genomic architecture, which will increase the efficiency of sorghum breeding programs. Major effect SNP loci, Sbi2393610 (PVE = 23.3%), Sbi10438246 (PVE = 35.2%), Sbi17789352 (PVE = 11.9%) and Sbi30169733 (PVE = 18.9%) on chromosomes 1, 3, 5 and 9 that showed strong association signals for PAWD, DTF, GY and PALH, respectively, are major findings of this study, which need to be further investigated. These findings provide insight into the genetic control of grain yield and agronomic traits, and after validation, the identified novel candidate genes may be used in genomics-led breeding for sorghum genetic improvement.

## Data availability statement

The datasets presented in this study can be found in online repositories. The names of the repository/repositories and accession number(s) can be found in the article/ Supplementary Material. All raw sequences are available at Sequence Reads Archive (SRA) database, BioProject PRJNA780262.

## Author contributions

All authors conceived and designed the experiment. ME conducted the field experiment. MG and ME mined and selected the SNP marker set for genotyping. ME analyzed the data with the guidance of MG. ME wrote the draft manuscript. MG, TF, AC, CH, KT, and AS revised the manuscript. All authors read the final version and approved the submission of the manuscript for publication.

## Funding

This research work was financially supported by the Swedish International Development Cooperation Agency (Sida) and Research and Training Grant awarded to Addis Ababa University and Swedish University of Agricultural Sciences (AAU-SLU Biotech; https://sida.aau.edu.et/index.php/biotechnology-phd-program/; accessed on July 10, 2022).

## Acknowledgments

The authors would like to thank the Swedish International Development Cooperation Agency (Sida) for financing this research, and Melkassa Agricultural Research Center and Ethiopia Biodiversity Institute for providing sorghum germplasm used in this study. Additionally, the authors would like to express their gratitude to Melkassa, agricultural research center and its Mehoni and Mieso sub-centers for allowing us to use their experimental stations, and for supplying tools and assisting with field activities. The authors thank Girma Gashu and Wubshet Mamo for their unreserved support in collecting phenotypic data.

## Conflict of interest

The authors declare that the research was conducted in the absence of any commercial or financial relationships that could be construed as a potential conflict of interest.

## Publisher’s note

All claims expressed in this article are solely those of the authors and do not necessarily represent those of their affiliated organizations, or those of the publisher, the editors and the reviewers. Any product that may be evaluated in this article, or claim that may be made by its manufacturer, is not guaranteed or endorsed by the publisher.
