# Supplementary figures and images for: Genome-wide analyses using multi-locus models revealed marker-trait associations for major agronomic traits in Sorghum bicolor
Source: Front Plant Sci. 2022 Oct 7;13:999692. doi: 10.3389/fpls.2022.999692 (PMC9585286; doi:10.3389/fpls.2022.999692)

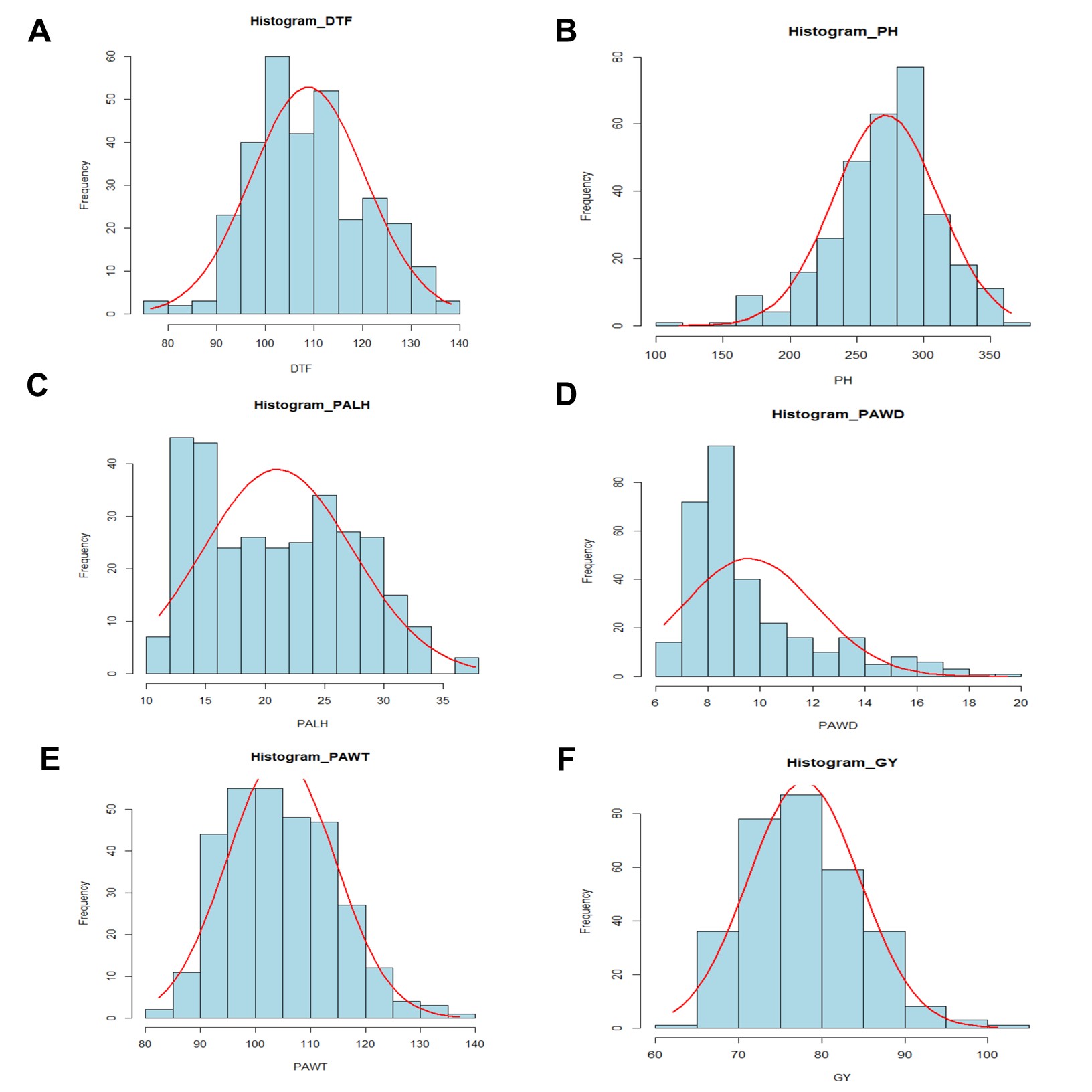

Supplement: Supplementary file 1 [file Image_1.jpeg]

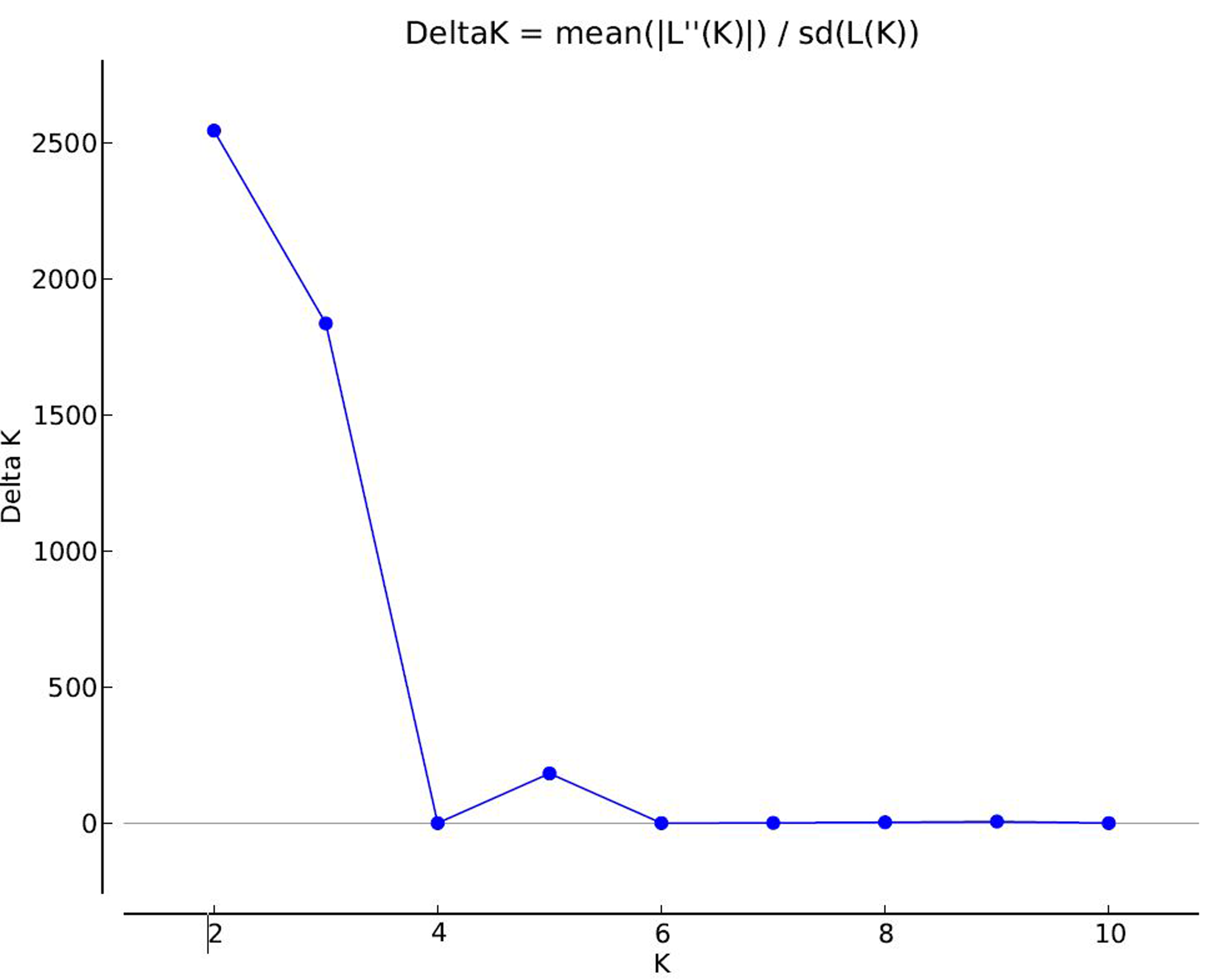

Supplement: Supplementary file 2 [file Image_2.tif]

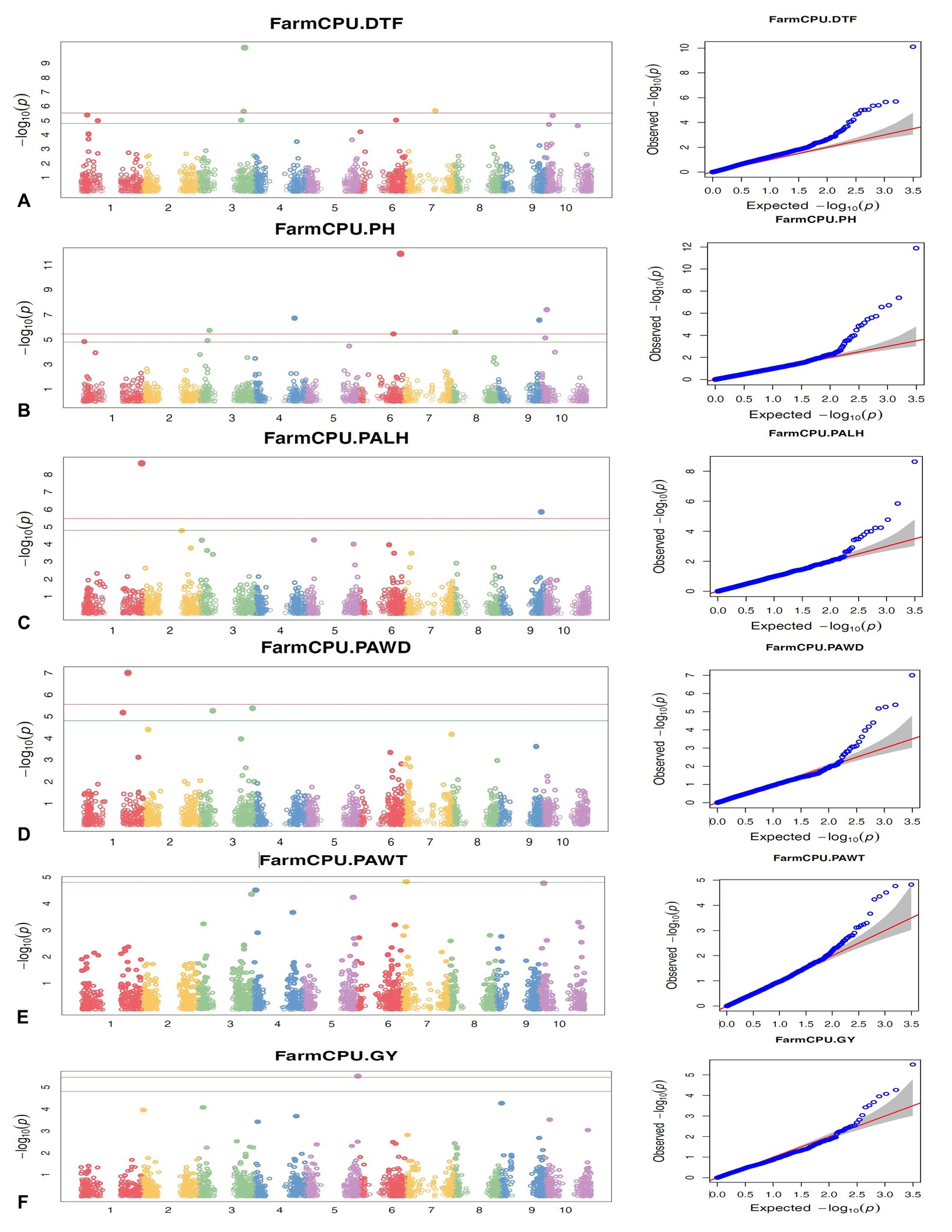

Supplement: Supplementary file 3 [file Image_3.jpeg]
